# The immune dysregulation landscape and dynamic regulation of competing endogenous RNAs in spinal cord injury

**DOI:** 10.3389/fimmu.2026.1774995

**Published:** 2026-05-07

**Authors:** Yuxuan Yao, Wenliang Guo, Xingyun Cai, Yuchang Gui, Jingzhi Yao, Yuting Lu, Yuxin Liang, Wenshu Wang, Zhibiao Tan, Jinxiang Zhang, Jianwen Xu

**Affiliations:** 1 Department of Rehabilitation Medicine, The First Affiliated Hospital of Guangxi Medical University, Nanning, China; 2 Guangxi Medical University, Nanning, Guangxi, China; 3 Department of Rehabilitation Medicine, Guigang City People's Hospital, Guigang, Guangxi, China; 4 Department of Joint Surgery and Geriatric Orthopedics, The Affiliated Hospital of Youjiang Medical University for Nationalities, Baise, China; 5 Guangxi Key Laboratory for Preclinical and Translational Research on Bone and Joint Degenerative Diseases, Baise, China

**Keywords:** ceRNA, immune infiltration, miRNA, molecular subtypes, spinal cord injury

## Abstract

**Background:**

Spinal cord injury (SCI) triggers a complex secondary injury cascade that critically limits neural repair. Although microRNAs have been implicated in post-SCI inflammation and neural regeneration, the key competing endogenous RNA (ceRNA) network regulating immune dysregulation remains unclear.

**Methods:**

Peripheral blood miRNA data from six SCI patients and six healthy controls were combined with peripheral blood mRNA expression profiles from the GEO dataset (GSE151371) to identify differentially expressed miRNAs and mRNAs. Characteristic miRNAs were screened using LASSO and SVM-RFE machine learning algorithms, with core miRNAs subsequently validated via receiver operating characteristic (ROC) analysis. Their target mRNAs were predicted and validated, enabling the construction of a ceRNA regulatory network. Concurrent immune infiltration analysis was performed. WGCNA was applied to the core mRNAs to define immune-related co-expression modules and conduct functional enrichment. Furthermore, SCI samples were classified into two molecular subtypes with distinct immune microenvironments. Finally, RT−qPCR was used to quantify the expression of core miRNAs in validation cohorts, and functional experiments (CCK-8 and Transwell assays) were performed in C8-D1A astrocytes to assess the roles of selected miRNAs in cell proliferation and migration.

**Results:**

Six differentially expressed miRNAs were identified in SCI patients: four upregulated (hsa-miR-3680-5p, hsa-miR-410-3p, hsa-miR-6883-3p, hsa-miR-4508) and two downregulated (hsa-miR-3685, hsa-miR-6798-3p). ROC analysis verified the robust diagnostic value of these core miRNAs, with AUC > 0.85. RT-qPCR validation confirmed these expression patterns in the blood of SCI patients, consistent with bioinformatics predictions, p < 0.05. Their common targets (KLHL3, PRKCH, BACH2, MAP2K6, SIPA1L2) showed opposite expression patterns. WGCNA revealed immune-related modules enriched in T/B cell receptor and MAPK signaling pathways. Immune infiltration analysis indicated increased neutrophils and monocytes, with decreased lymphocytes. Notably, functional assays demonstrated that miR-3680-5p, miR-6798-3p, and miR-6883-3p did not directly affect astrocyte proliferation but significantly modulated astrocyte migration when co-cultured with macrophages, suggesting their involvement in astrocyte-macrophage crosstalk during SCI repair.

**Conclusion:**

We constructed an immune-related ceRNA network in SCI, identifying six core miRNAs that regulate immune pathways and astrocyte-macrophage interactions. These findings provide new insights into SCI pathogenesis and potential therapeutic targets.

## 1 Introduction

SCI is a severe traumatic disease of the central nervous system, characterized by a high disability rate and low cure rate, with its global incidence showing an annual upward trend (1, 2). It has been reported that there are 759,302 patients with traumatic spinal cord injury in China, with 66,374 new cases added each year (3). The repair of neurological function after SCI is restricted by multiple factors, among which the dynamic changes of the SCI microenvironment are the core link determining the efficiency of nerve regeneration. This microenvironment comprises components such as nerve cells, glial cells, immune cells, and extracellular matrix. After injury, pathological changes, including nerve cell apoptosis, activation of astrocytes to form glial scars, massive release of inflammatory factors, and vascular regeneration disorders, occur, which collectively constitute a “pathological microenvironment” that inhibits nerve repair and becomes a key bottleneck in SCI treatment (4). Therefore, it is essential to explore the molecular mechanisms of SCI progression and identify specific analytical targets that may lead to effective treatment of SCI.

To identify such specific analytical targets, researchers have increasingly shifted their focus from traditional protein-coding genes to complex post-transcriptional regulatory networks. In recent years, the regulatory role of non-coding RNAs in tissue repair and injury has gradually become a research focus. Among them, the ceRNA regulatory network, with its regulatory characteristics of “multi-molecule coordination and multi-pathway crosstalk”, provides a new perspective for deciphering the mechanism of SCI microenvironment remodeling. The ceRNA network utilizes molecules such as lncRNAs and circRNAs as “molecular sponges” to competitively sequester miRNAs. This sequestration relieves the transcriptional repression of downstream target genes by miRNAs, thereby regulating biological processes including cell proliferation, differentiation, apoptosis, and inflammatory responses (5, 6). Existing studies have confirmed that the ceRNA network can promote tissue repair by regulating the injury microenvironment in diseases such as brain injury and peripheral nerve injury (7, 8). Notably, recent high-throughput studies have begun to map the landscape of ceRNA networks in various SCI contexts. For instance, Huang et al. identified the AABR07041411.1/miR-125a-5p/Slc4a7 axis as a key regulator of neuroinflammation (9), while Liu et al. and Xia et al. independently characterized pyroptosis-related ceRNA modules involving Casp1 and GSDMD (10, 11). However, in the field of SCI, there remain numerous unsolved issues regarding its specific regulatory mechanisms: On one hand, the key ceRNA molecules (especially tissue-specific lncRNAs/circRNAs) in the SCI microenvironment have not yet been systematically identified; on the other hand, the molecular interaction mechanisms underlying how the ceRNA network precisely regulates core pathological processes of the microenvironment, such as glial scar formation, inflammation resolution, and directed differentiation of neural stem cells, remain unclear. Therefore, exploring the mechanism of ceRNA in SCI may provide a new approach to understanding its pathophysiological processes and potential treatments.

In this study, we employed bioinformatics techniques to identify biomarkers associated with the progression of SCI. We constructed ceRNA networks to investigate key mRNA-immune cell interactions and performed quantitative RT-qPCR analysis on core genes. Our findings provide additional insights into the molecular and immunological framework that explains SCI pathology.

## 2 Materials and methods

### 2.1 Sample collection and miRNA sequencing

We conducted genome-level miRNA sequencing in peripheral blood mononuclear cells (PBMCs) from six SCI patients and six age, gender, and race-matched healthy controls. A total of 2.5 mL of each individual’s blood was directly collected into PAXgene^®^ Blood RNA Tubes (BD Biosciences, USA, catalog No.762165). Following the manufacturer’s protocol, the tubes were gently inverted 8 to 10 times, left at room temperature for at least 2 hours, and stored at 4 °C. The collected samples were transported to the GBRC Laboratory for further processing, following the PAXgene Blood miRNA Kit manual (PreAnalytiX, Switzerland, catalog No. 763134). After collection, the RNA samples were stored at -80 °C until further processing. RNA concentration and purity were measured using a Qubit 4 fluorometer (Thermo Fisher Scientific, USA) and Qiaxpert (Qiagen, USA). Library preparation was performed using the Ion Total RNA-Seq Kit v2 (Thermo Fisher Scientific, USA, catalog No. 4479789), which first enriched total RNA to obtain miRNA before library construction, following the manufacturer’s instructions. The Ion 540™ Chip Kit (Thermo Fisher Scientific, USA, catalog No. A27765) was utilized for subsequent sequencing on the Ion S5™ system (Thermo Fisher, USA). Sequencing platform reading data was collected at the end of the miRNA sequencing analysis. The FASTQC program, quality filters, and Trimmomatic application for cutting operations software were used to remove unwanted data (12).

### 2.2 Differential expression analysis of miRNAs and mRNAs

Based on miRNA-seq, we obtained miRNA expression data from 6 SCI samples and six standard control samples. Differential expression analysis was conducted using the “DESeq2” and “tidyverse” packages in R to identify differentially expressed miRNAs (DEmiRNAs) between SCI samples and control samples. A miRNA was considered a significant DEmiRNA if it met the criteria of a p-value < 0.05 and an absolute value of log2 fold change (log2FC) ≥ 1. Positively regulated miRNAs were selected as upregulated candidate miRNAs, while downregulated candidate miRNAs were derived from negatively regulated miRNAs. For result visualization, volcano plots and heatmaps were generated using the “ggplot” and “pheatmap” packages in R. The GSE151371 dataset was downloaded from the Gene Expression Omnibus (GEO) database (https://www.ncbi.nlm.nih.gov/geo/). This dataset comprises mRNA expression data in peripheral blood cells from 38 samples of SCI and 20 non-SCI samples. Differential expression analysis was performed using the “limma” and “tidyverse” packages in R to identify differentially expressed mRNAs (DEMs) between SCI samples and control samples. An mRNA was defined as a significant DEM when it satisfied the thresholds of a p-value < 0.05 and an absolute value of log2 fold change (log2FC) ≥ 1. Therefore, among SCI-related DEMs, positively regulated mRNAs were chosen as upregulated candidate mRNAs, whereas downregulated candidate mRNAs were selected from the set of negatively regulated mRNAs. Volcano plots and heatmaps for visualizing the results were generated using the “ggplot” and “pheatmap” packages in R.

### 2.3 Pathway enrichment analysis of DEMs

To better understand the biological functions of DEMs, we performed enrichment analysis, including Gene Ontology (GO) and Kyoto Encyclopedia of Genes and Genomes (KEGG) pathway analyses with all DEMs as the background set, using the “limma”, “clusterProfiler”, and “org.Hs.eg.db” packages. The top terms with a p-value < 0.05 were considered statistically significant.

### 2.4 Feature selection using two machine learning algorithms

To select the most discriminative miRNA features, we applied two machine learning algorithms to screen core miRNAs base on DEmiRNAs, utilizing least absolute shrinkage and selection operator (LASSO) regression and support vector machine-recursive feature elimination (SVM-RFE) methods to identify features that distinguish SCI patients from controls. The two algorithms were applied in a cross-validation manner to increase the reliability of the selected features. These processes were performed using the R packages “glmnet” and “kernlab”, with the random seed set to 123 for reproducibility. Ten-fold cross-validation was implemented on the training set to determine the core miRNAs based on the minimum error values and to prevent overfitting. Following this, a visualization of the overlapping miRNAs across the different algorithms was performed using the web-based application (http://jvenn.toulouse.inra.fr/).

### 2.5 Screening and expression validation of SCI biomarkers

To further screen the core miRNAs in SCI, we performed validation using ROC curve analysis using the pROC package to evaluate the diagnostic efficacy of each core miRNA independently. We use the original dataset as the validation dataset. The area under the ROC curve (AUC) value approaching 1 indicates that the screened miRNAs possess reasonable specificity and sensitivity, suggesting higher accuracy as potential biomarkers for the disease. Furthermore, we selected miRNAs with an AUC > 0.8 as core miRNAs for subsequent studies, validated their expression levels in both the SCI and control groups, and visualized the results using violin plots.

### 2.6 Prediction of target mRNAs for core miRNAs and validation of hub mRNA expression levels

Using the miRWalk online platform, we predicted the target mRNAs for each of the six core miRNAs with a binding score threshold > 0.8. Subsequently, we compared these target mRNAs with the previously identified DEMs to obtain common mRNAs. These six sets of common mRNAs were input into an online Venn diagram tool (http://jvenn.toulouse.inra.fr/) to identify intersection mRNAs, which were designated as hub mRNAs. Expression levels of these hub mRNAs were extracted from the GSE151371 dataset, and violin plots were generated using the ggplot2 package in R to visualize the expression differences of hub mRNAs between the SCI group and the control group.

### 2.7 Construction of the CeRNA network

LncRNAs/circRNA acting as upstream regulators of miRNAs were identified using StarBase v2.0 with CLIP-data ≥ 1 and lncBase v3. Downstream target mRNAs were predicted using MiRWalk (http://mirwalk.umm.uni-heidelberg.de/) as described above (13). Since both databases contain experimentally validated miRNA-target interactions, we ensured the acquisition of robust and consistent lncRNA/circRNA-miRNA-mRNA expression pairs for constructing the ceRNA network. Sankey diagrams were generated using the R package “ggplot2” to visualize the network. Additionally, the interactions between core miRNAs and hub mRNAs were illustrated with the assistance of Cytoscape (Version 3.10.3).

### 2.8 Elucidation of SCI-associated molecular subtypes and differences between subtypes

Consensus clustering analysis was performed using the “ConsensusClusterPlus” package to identify subtypes of SCI. The parameters for this analysis were set as follows: sample resampling rate of 80%, number of resamplings at 100, and maximum number of clusters at 9. The optimal cluster count was determined by analyzing the cumulative distribution function (CDF), consensus matrix, and changes in the area under the CDF curve. The results of molecular typing were visualized using the heatmap package. We conducted differential analysis on SCI subtype data using the “limma” package and visualized the results with heatmaps and volcano plots. mRNAs among the top 10 in terms of |logFC| values were selected as differential mRNAs that also had a p-value < 0.05 for further validation, and their expression levels were visualized using violin plots.

### 2.9 Immune cell infiltration in the SCI microenvironment

To explore the potential association between hub mRNA expression and immune infiltration in SCI, this study employed partial deconvolution and linear least squares regression analyses. The CIBERSORT algorithm accurately characterized the composition of immune cells in tissues by integrating the expression data of 547 genes. Based on the sample data of 22 infiltrating immune cell types from the GSE151371 project, this study used the CIBERSORT algorithm to quantitatively analyze gene expression, with a statistical significance threshold set at p < 0.05. The R packages “ggcorrplot” and “ggplotify” were used to determine the correlations between immune cells in GSE151371, and a heatmap was constructed for visualization. Differences in immune cell abundance between the SCI group and the control group were displayed using grouped boxplots.

### 2.10 Correlation analysis and validation between hub mRNAs and immune cells

The Spearman test was used to investigate the correlation between immune infiltration and hub mRNAs. A correlation lollipop plot was constructed using the “ggplot2” package to visualize the correlations between hub mRNAs and 22 immune cell types, with p < 0.05 considered statistically significant. Additionally, scatter plots were used to clarify the correlation coefficients between hub mRNAs and immune cells.

### 2.11 Correlation analysis between SCI subtypes and immune cells

The CIBERSORT algorithm was used to perform immune infiltration analysis of 22 immune cell types in 38 SCI samples from the GSE151371 dataset, and violin plots were employed to clarify differences in immune cell infiltration between SCI subtypes. Subsequently, Spearman correlation analysis was conducted to examine the correlations between 10 differential mRNAs and immune cells, with correlation heatmaps visualized using the “ggplot” package.

### 2.12 WGCNA and pathway enrichment analysis of identified modules

WGCNA was performed to place the differentially expressed genes within a systems-level co-expression framework and to identify modules enriched for genes related to immune cells. After calculating Pearson correlation coefficients to construct the co-expression matrix, a soft-thresholding power of 20 (R^2^ = 0.80) was selected based on scale-free topology. The adjacency matrix was converted into a topological overlap matrix (TOM), and hierarchical clustering was used to identify gene modules with a minimum size of 50 genes and a mergeCutHeight of 0.1. Firstly, the correlation between each module and the sample trait (Control and SCI) was assessed. Then, the correlation between the module eigengenes and 22 immune cell types was determined using Pearson’s correlation test, considering a p-value of < 0.05 was considered statistically significant.

### 2.13 RT-qPCR

To validate the expression levels of the identified core miRNAs, total RNA was carefully isolated from the blood of patients and healthy individuals using TRIzol reagent (Solarbio, 15596-026, China). Subsequently, miRNA and other RNA molecules were reverse-transcribed into cDNA using the Mir-X miRNA first-strand synthesis kit (TaKaRa Bio, USA). To measure gene expression levels, we performed RT-qPCR using the Takara TB Green^®^ Fast qPCR Mix kit (Takara, RR430A, China) in conjunction with the LightCycler 7500 system. Each sample underwent three replicate experiments to ensure the robustness of the data. Quantitative analysis of miRNA expression levels was performed using the 2−ΔΔCt method.

### 2.14 Transfection

To achieve stable and efficient knockdown of specific miRNAs in C8-D1A cells, a lentiviral-mediated RNA interference approach was employed. Recombinant lentiviruses carrying short hairpin RNA (shRNA) sequences, along with a scrambled shRNA sequence serving as a negative control (LV-NC), were constructed. The shRNA sequences were cloned into the pLKO.1-puro lentiviral vector, and the resulting constructs were co-transfected into HEK293T cells with packaging plasmids (psPAX2 and pMD2.G) using a calcium phosphate precipitation method. Viral supernatants were collected at 48 and 72 h post-transfection, filtered through a 0.45 μm filter, and concentrated by ultracentrifugation. For transduction, C8-D1A cells were seeded in 6-well plates at a density of 5 × 10^4^ cells per well and cultured overnight. The following day, cells were incubated with the concentrated lentiviral particles in the presence of 8 μg/mL polybrene for 24 h. Subsequently, the transduction medium was replaced with fresh complete DMEM, and stably transduced cells were selected by treatment with 2 μg/mL puromycin for 72 h.

### 2.15 Transwell assay

Cell migration was assessed using a 24-well Transwell system with polycarbonate membrane inserts (8 μm pore size; Corning, NY, USA). The macrophages (5 × 10^4^ cells/well) were seeded in the lower chamber in 600 μL of DMEM containing 10% FBS and allowed to adhere overnight. C8-D1A cells were transfected as described above. At 48 h post-transfection, cells were harvested, washed with PBS, and resuspended in serum-free DMEM. A total of 5 × 10^4^ C8-D1A cells in 200 μL of serum-free medium were seeded into the upper chamber. The co-culture system was then incubated at 37 °C in a 5% CO_2_ atmosphere for 24 h. After incubation, non-migrated cells on the upper surface of the membrane were gently removed with a cotton swab. The migrated cells attached to the lower surface were fixed with 4% paraformaldehyde for 20 min and stained with 0.1% crystal violet for 15 min. Stained cells were visualized and imaged under an inverted microscope (Olympus, Japan) at ×100 magnification. For quantification, five randomly selected fields per well were counted, and the average number of migrated cells was calculated. Each experiment was performed in triplicate and repeated independently three times.

### 2.16 CCK-8 assay

To determine whether these core miRNAs directly affect astrocyte proliferation, cell proliferation was evaluated using the Cell Counting Kit-8 (CCK-8; Dojindo, Kumamoto, Japan) following the manufacturer’s protocol. C8-D1A cells were seeded into 96-well plates at a density of 3 × 10³ cells per well in 100 μL of complete DMEM and cultured overnight to allow attachment. After transfection, cells were incubated for 24, 48, and 72 h. At each time point, 10 μL of CCK-8 solution was added to each well, followed by incubation at 37 °C for an additional 2 h in the dark. Absorbance was measured at 450 nm using a microplate reader (Bio-Rad, Hercules, CA, USA). Cell viability was expressed as a percentage relative to the control group (set as 100%). All experiments were performed with five replicate wells per condition and independently repeated three times to ensure reproducibility.

### 2.17 Statistical analysis

All bioinformatic and statistical evaluations were performed using R software (version 4.4.2). Differences between groups were assessed using an unpaired t-test or one-way ANOVA. Data are presented as the mean ± standard deviation (SD). Correlations were evaluated by Spearman correlation analysis. Results with two-sided p values < 0.05 were considered to be statistically significant unless otherwise specified above.

## 3 Results

### 3.1 Identification of DEmiRNAs and DEMs

**Figure 1:**
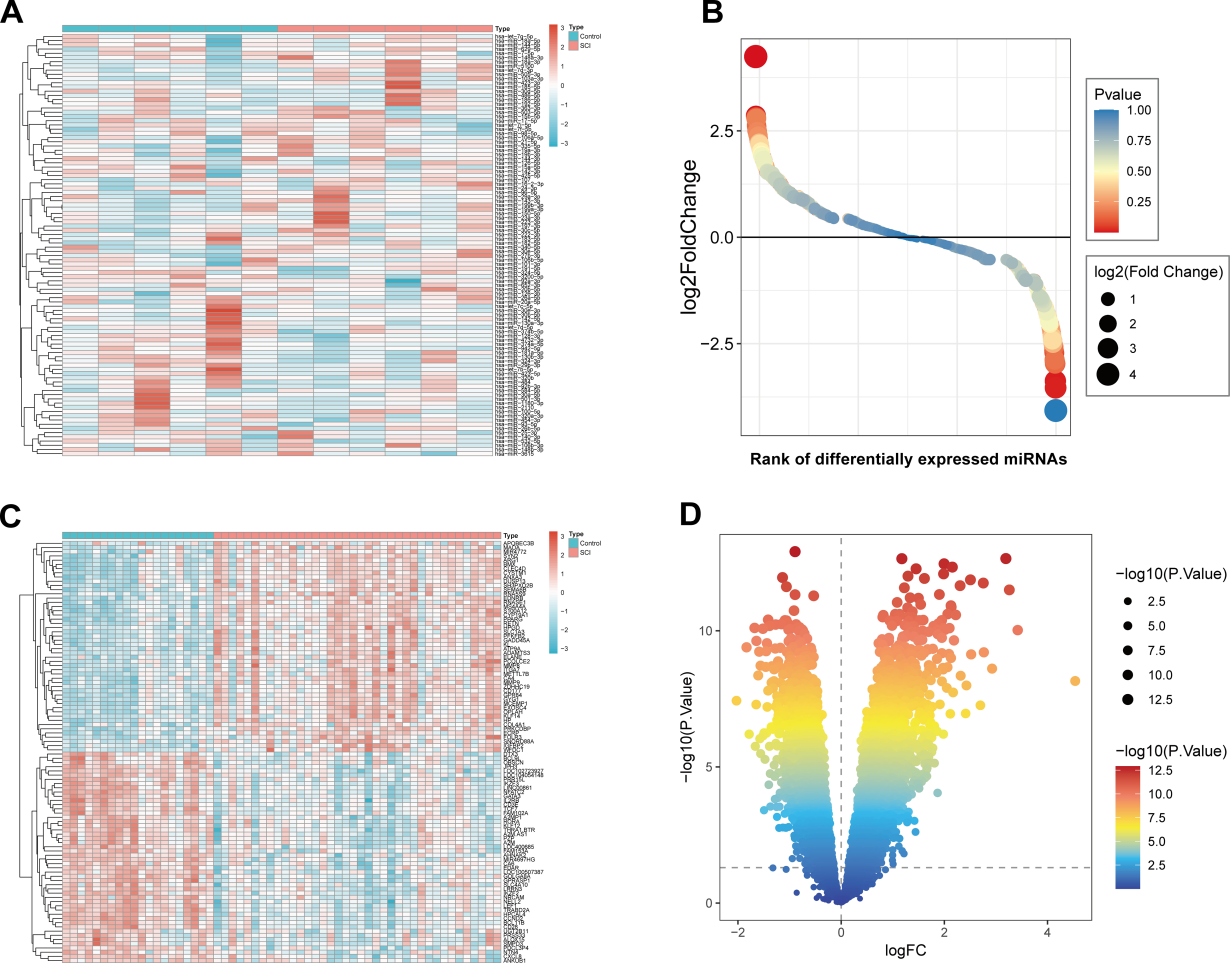
DEmiRNAs identification. **(A)** Heatmap displays the differences in miRNA expression between control samples and treated samples. **(B)** The volcano plot shows the significance and fold change of differentially expressed miRNAs. **(C)** Heatmap displays the differences in mRNA expression between control samples and treated samples. **(D)** The volcano plot shows the significance and fold change of differentially expressed mRNAs.

We performed a differential analysis of miRNA sequencing data from blood samples of six SCI patients and six healthy individuals. The results revealed significant differences in gene expression between control samples and samples from SCI patients. Specifically, a heatmap presented the expression levels of DEmiRNAs between the two groups (**Figure 1A**). As observed in the heatmap, some miRNAs show specific trends of high or low expression between SCI and normal samples. Volcano plots were further used to visualize the significance and fold changes of these DEmiRNAs (**Figure 1B**), facilitating the identification of significantly upregulated and downregulated miRNAs. The distribution characteristics of these miRNAs showed that a large number of DEmiRNAs were concentrated in the upper-left and lower-right regions of the volcano plot. In this study, a total of 2107 miRNAs were detected. Based on the criteria of p < 0.05 and |log2FC| > 1, 45 DEmiRNAs were identified, including 36 significantly upregulated and 9 significantly downregulated miRNAs. Differential analysis of the GSE151371 dataset demonstrated significant differences in mRNA expression between control samples and SCI samples. Specifically, a heatmap displayed the expression levels of DEMs between the two groups (**Figure 1C**). It could be seen from the heatmap that mRNAs highly expressed in control samples were generally downregulated in SCI samples. In contrast, mRNAs with low expression in control samples usually showed an upregulation trend in SCI samples. Volcano plots were used to visualize the significance further and fold changes of these DEMs (**Figure 1D**), which helped identify significantly upregulated and downregulated mRNAs. The distribution characteristics of these mRNAs indicated that a large number of DEMs were concentrated in the upper-left and upper-right regions of the volcano plot. In this study, 17,500 mRNAs were analyzed, and 1156 DEMs were identified.

### 3.2 Gene function enrichment of DEMs

**Figure 2:**
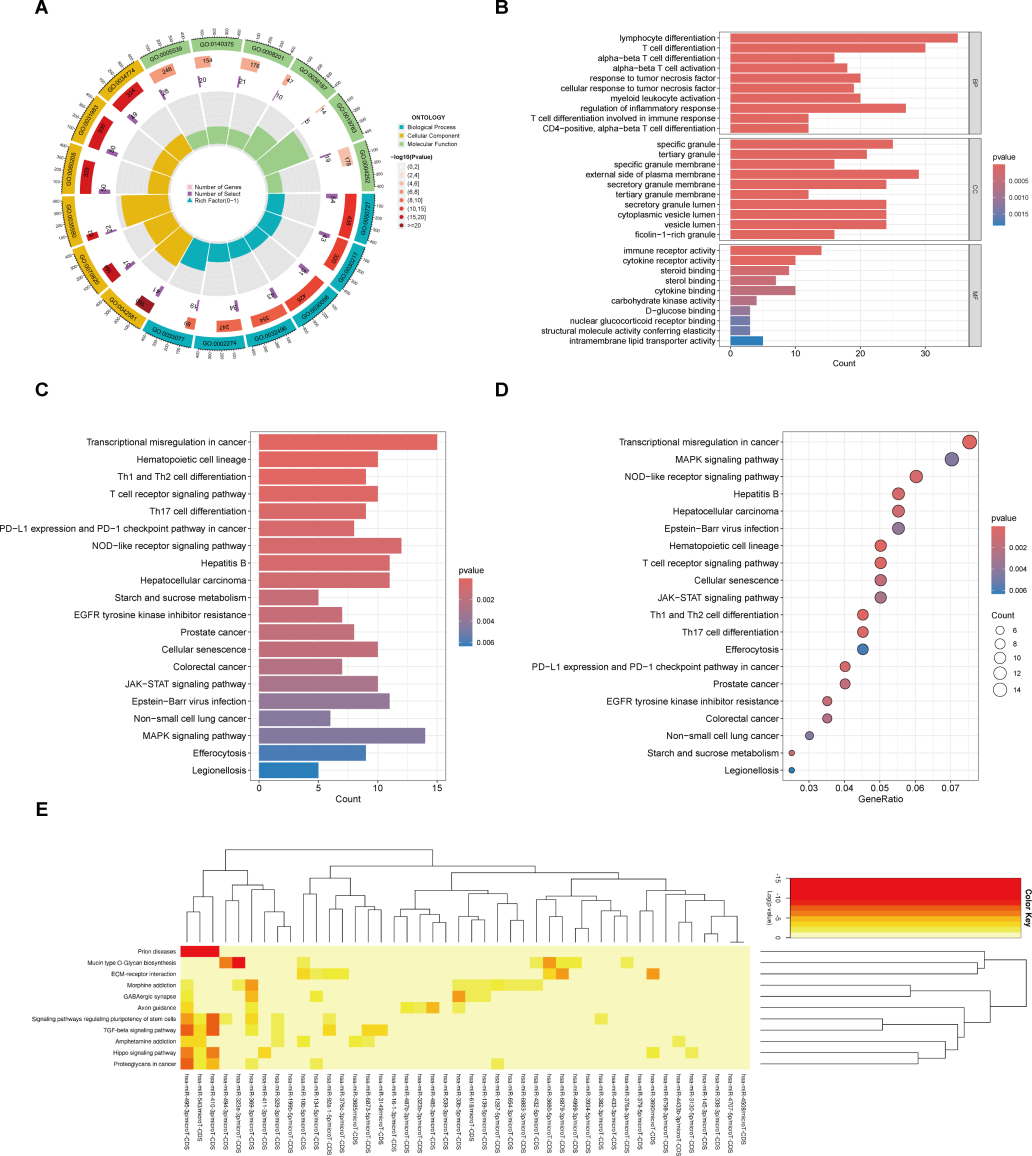
The GO and KEGG functional enrichment of DEmiRNAs and DEMs. **(A)** The circle plot of GO enrichment of DEMs. **(B)** The bar plot of GO enrichment of DEMs. **(C)** The bar plot of KEGG enrichment of DEMs. **(D)** The bubble plot of KEGG enrichment of DEMs. **(E)** The KEGG enrichment of DEmiRNAs.

Following GO and KEGG pathway analyses of DEMs, we found that most DEMs were predominantly enriched in inflammation-related pathways and immune cell-associated pathways. In the biological process (BP) category of the GO analysis, the results notably highlighted that these DEMs were primarily involved in pathways such as “T cell differentiation”, “response to tumor necrosis factor”, and “regulation of inflammatory response”. In the molecular function (MF) category, DEMs were enriched in pathways including “cytokine receptor activity” and “immune receptor activity” (**Figures 2A, B**). Meanwhile, KEGG analysis results demonstrated that DEMs were mainly associated with pathways such as “T cell receptor signaling pathway” and “Th1 and Th2 cell differentiation” (**Figures 2C, D**). Additionally, the heatmap of miRNA enrichment analysis revealed that “TGF-β signaling pathway” and “Hippo signaling pathway” were among the most prominently enriched pathways, further supporting the involvement of immune-inflammatory regulatory mechanisms (**Figure 2E**).

### 3.3 Identification of signature miRNAs for SCI

**Figure 3:**
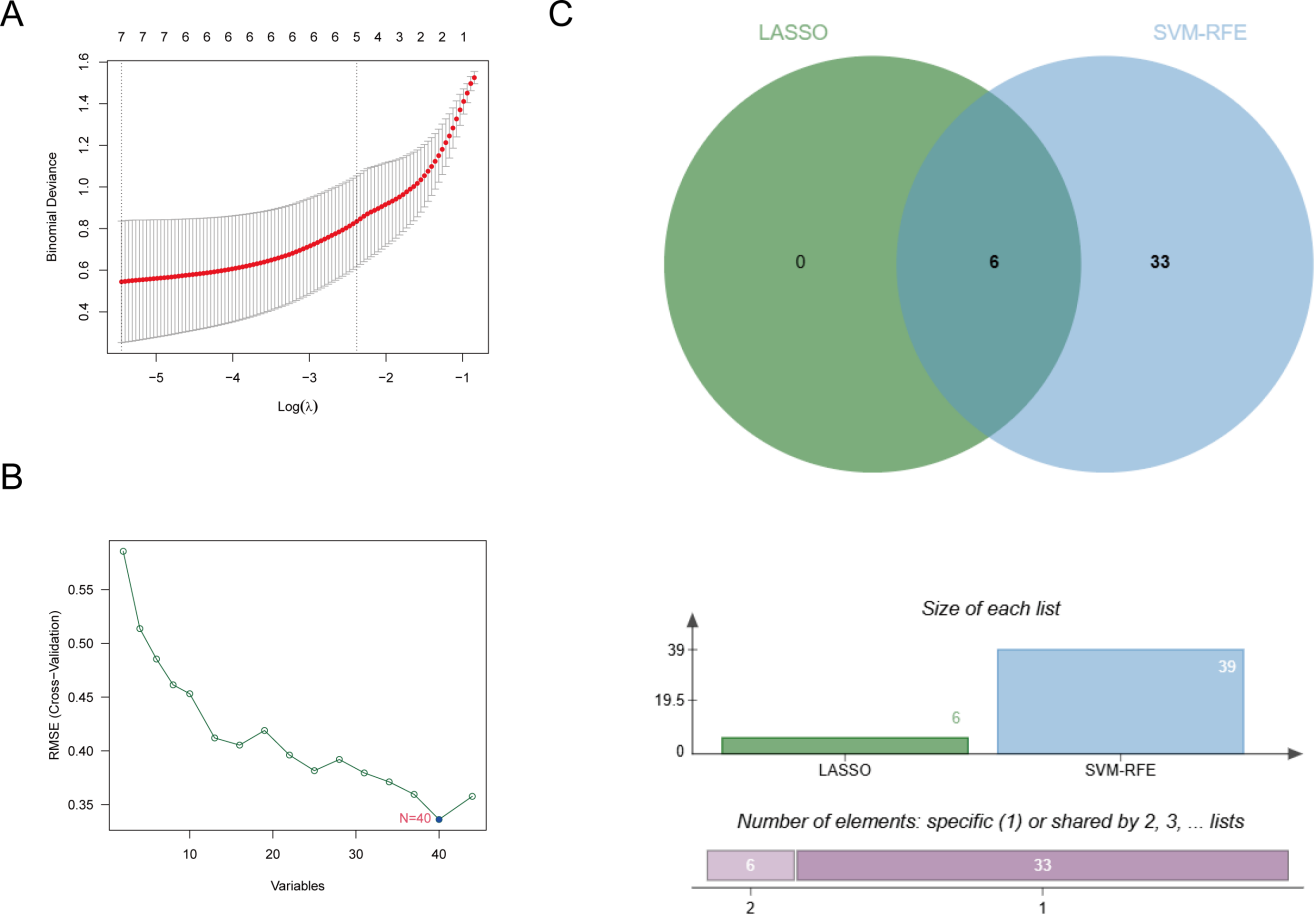
Identification of core miRNAs for SCI. **(A)** LASSO approach identification. **(B)** SVM-RFE algorithm filtering. **(C)** Venn diagram showing algorithm intersections.

Multiple machine learning approaches were employed to identify signature miRNAs for SCI. First, six signature miRNAs were screened using the optimal lambda (λ) value in LASSO logistic regression (**Figure 3A**). Subsequently, the screening was further refined using the SVM-RFE algorithm, where the optimal targets were selected based on the lowest root mean square error and highest accuracy, resulting in the identification of 39 signature miRNAs (**Figure 3B**). Finally, the optimal signature miRNAs commonly identified by both methods were prioritized, leading to the determination of 6 miRNAs, namely hsa-miR-3680-5p, hsa-miR-3685, hsa-miR-410-3p, hsa-miR-4508, hsa-miR-6798-3p, and hsa-miR-6883-3p, for subsequent analyses (**Figure 3C**).

### 3.4 Identification of core mRNAs and expression level verification

**Figure 4:**
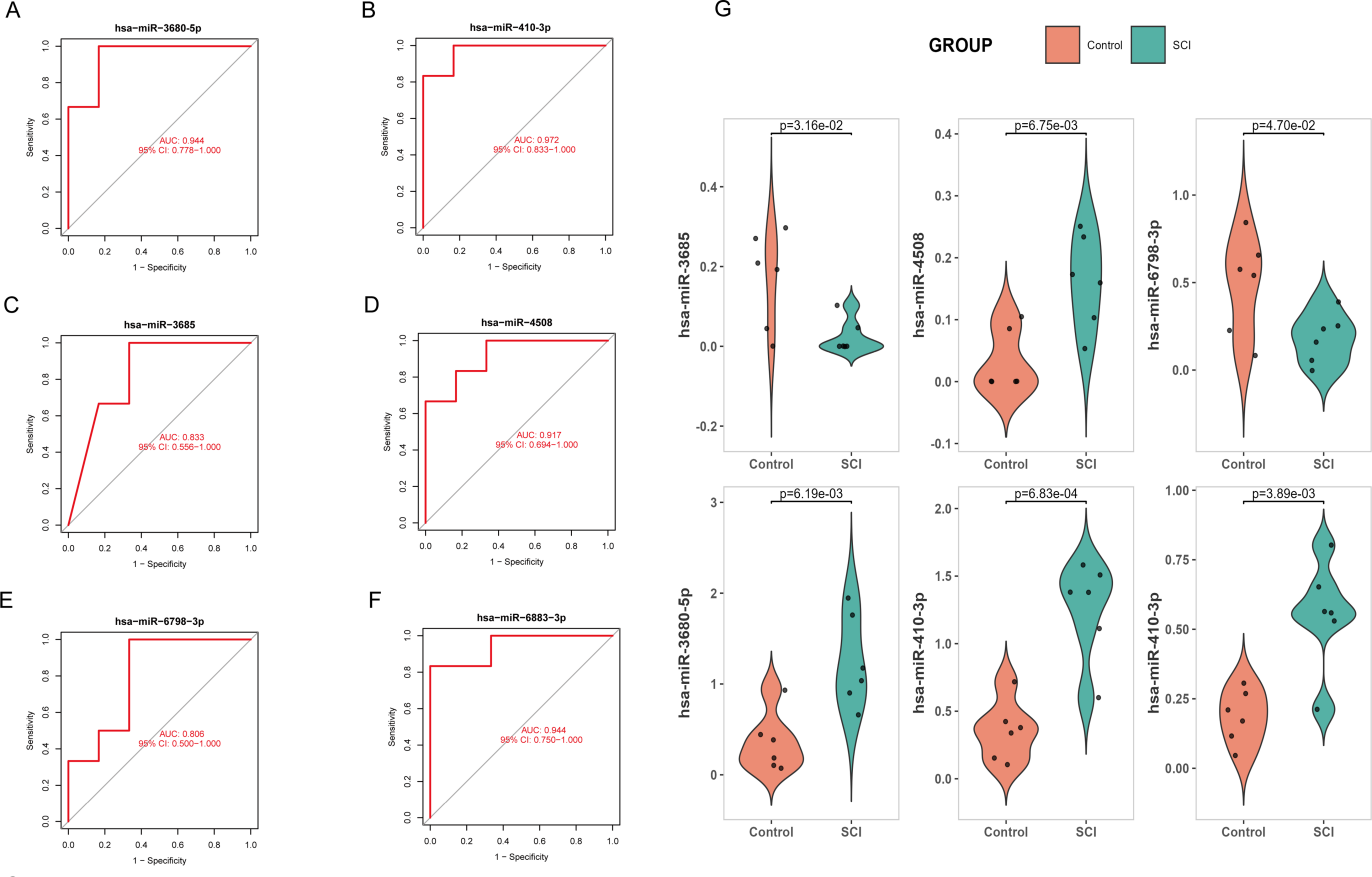
Logistic regression model underwent ROC analysis. **(A)** ROC evaluation of the hsa-miR-3680-5p. **(B)** ROC evaluation of the hsa-miR-410-3p. **(C)** ROC evaluation of the hsa-miR-3685. **(D)** ROC evaluation of the hsa-miR-4508. **(E)** ROC evaluation of the hsa-miR-6798-3p. **(F)** ROC evaluation of the hsa-miR-6883-3p. **(G)** Expression of 3 miRNAs in the control group and SCI group.

To further confirm the core miRNAs in SCI, we plotted the ROC curve for each signature miRNA and calculated the AUC. We used the original dataset as the validation set. Six of these miRNAs exhibited an AUC > 0.8, demonstrating satisfactory diagnostic efficacy (**Figures 4A–F**). To enhance reliability and accuracy and facilitate subsequent analyses, we selected six miRNAs with an AUC > 0.8, namely hsa-miR-3680-5p, hsa-miR-410-3p, hsa-miR-6883-3p, hsa-miR-3685, hsa-miR-4508 and hsa-miR-6798-3p, as core miRNAs. Subsequently, we verified the expression levels of these six core miRNAs. It was observed that their expression of hsa-miR-3680-5p, hsa-miR-410-3p, hsa-miR-6883-3p, and hsa-miR-4508 in the SCI group was significantly higher than that in the control group, whereas hsa-miR-3685 and hsa-miR-6798-3p showed the opposite trend (**Figure 4G**).

### 3.5 Identification of hub mRNAs and construction of the ceRNA network

**Figure 5:**
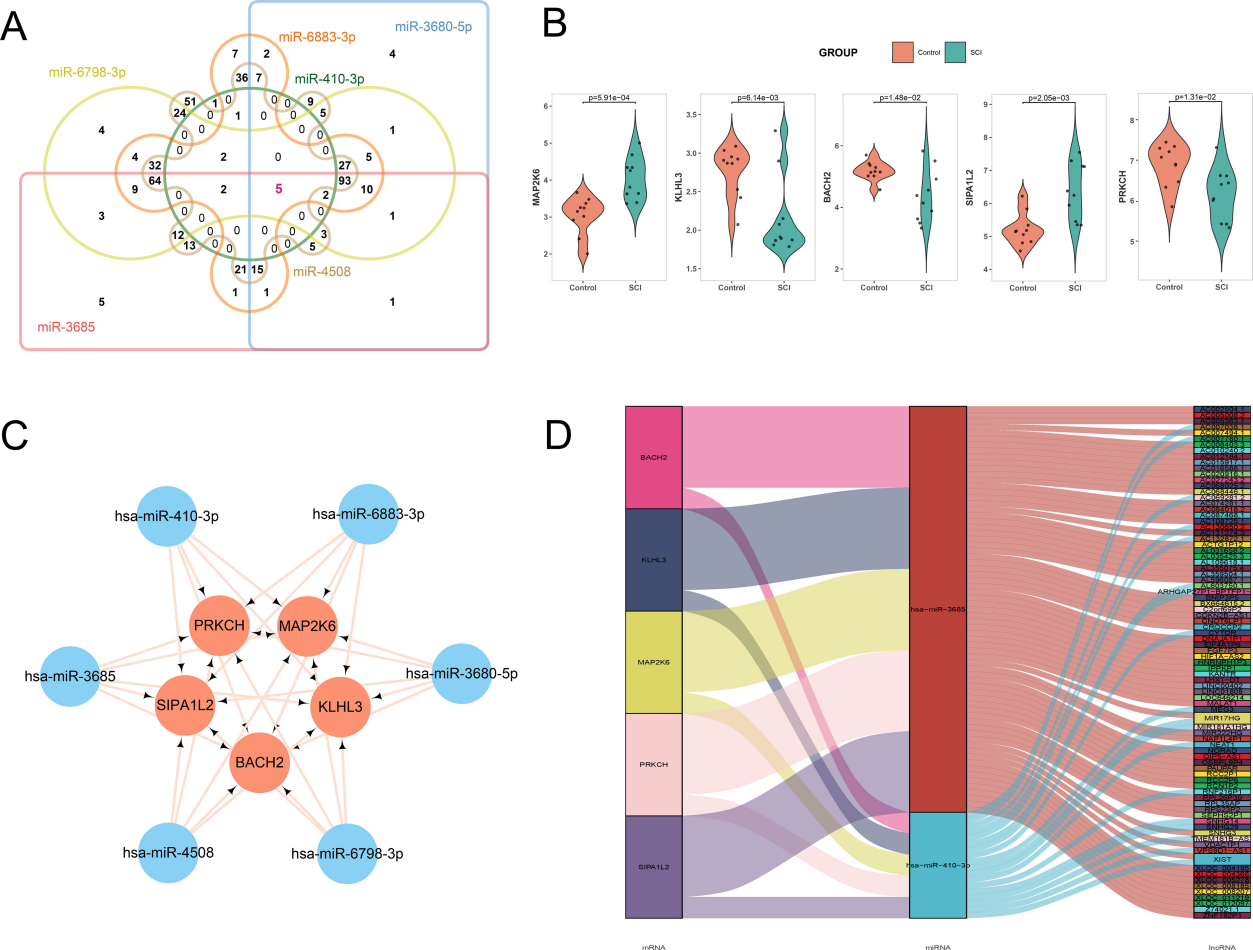
Identification of the hub mRNA and Construction of ceRNA networks. **(A)** Intersection of hsa-miR-3680-5p, hsa-miR-410-3p, hsa-miR-6883-3p, hsa-miR-3685, hsa-miR-4508 and hsa-miR-6798-3p target mRNAs. **(B)** Expression of 5 mRNAs in the control group and SCI group. **(C)** The interaction between 2 core miRNAs and 5 hub mRNAs. **(D)** Sankey plot showing the mRNA-miRNA-lncRNA expression pairs.

Further, we predicted the target mRNAs of the core miRNAs. To explore the mRNAs functioning in SCI, we took the intersection of the predicted target mRNAs and differentially expressed DEMs, obtaining 470 common target mRNAs: 346 target mRNAs for hsa-miR-6883-3p, 13 for hsa-miR-410-3p, 196 for hsa-miR-3680-5p, 266 for hsa-miR-3685, 427 for hsa-miR-4508 and 308 for hsa-miR-6798-3p, respectively. Subsequently, we intersected these target mRNAs and found that five mRNAs were shared as common targets of the six miRNAs, designated as hub mRNAs: MAP2K6, PRKCH, KLHL3, SIPA1L2 and BACH2 (**Figure 5A**). We also verified the expression levels of these five hub mRNAs. The results showed that MAP2K6 and SIPA1L2 were upregulated in the SCI group, while both KLHL3, BACH2 and PRKCH were downregulated in the SCI group (**Figure 5B**). Among them, we selected the five mRNAs for further analysis and constructed a competing ceRNA network, precisely identifying the upstream lncRNAs/circRNA of core miRNAs and the interaction nodes between hub mRNAs and miRNAs. In this study, the core component of the ceRNA network consists of the mutual regulatory interactions between the six core miRNAs and five hub mRNAs (**Figure 5C**). To further refine the ceRNA network, we predicted lncRNAs and circRNAs (**Supplementary 1**) involved in regulating the core miRNAs. Among the core miRNAs, only hsa-miR-410-3p was found to have upstream regulatory lncRNAs and circRNAs, which exhibited the highest reliability. Through these interactions, we identified four lncRNAs, NEAT1, SNHG14, SNHG29, and XIST, that serve as common upstream lncRNAs for both hsa-miR-3680-5p and hsa-miR-410-3p (**Figure 5D**).

### 3.6 Molecular subtyping in SCI

**Figure 6:**
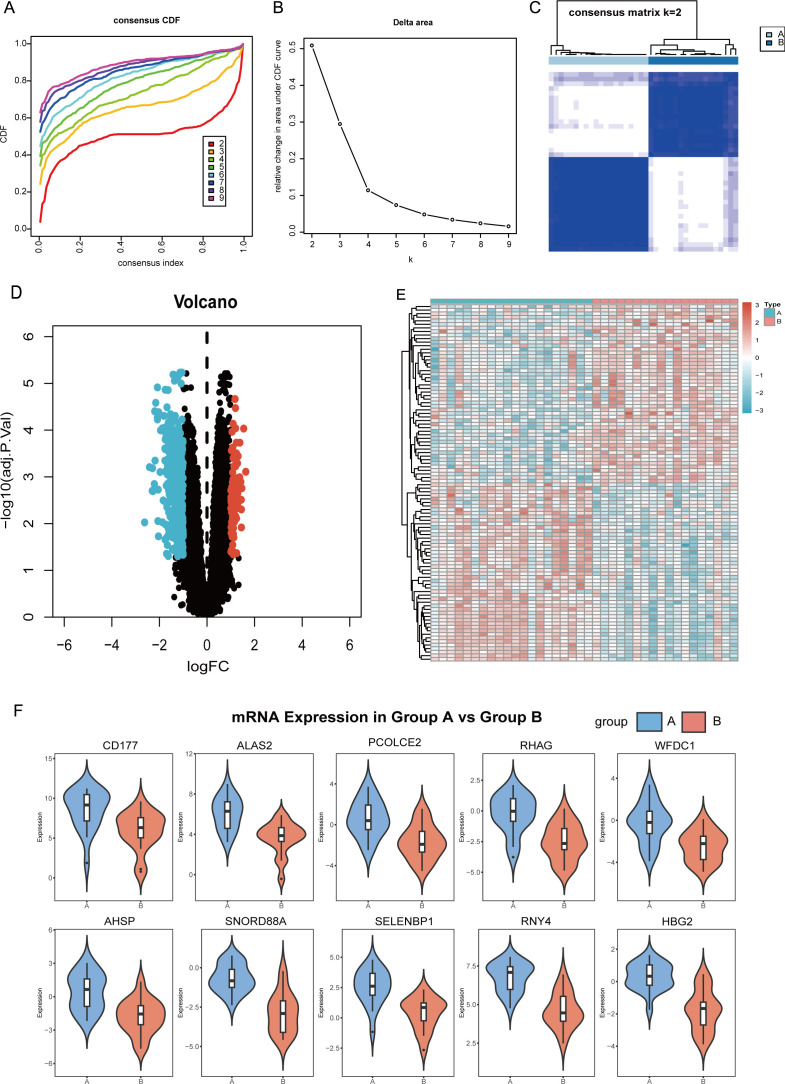
Molecular subtypes and expression of ten different mRNAs in SCI. **(A)** Cumulative distribution function (CDF) curve. **(B)** Area variation under the CDF curve. **(C)** Cluster heatmap for SCI molecular subtype A and subtype B. **(D)** The volcano plot shows the significance and fold change of differentially expressed mRNAs. **(E)** Heatmap displays the differences in mRNA expression between control samples and treated samples. **(F)** Expression difference in 10 significantly mRNAs between Cluster A and Cluster B.

To identify the intrinsic immune subtypes of SCI, we utilized the expression profiles of 1155 DEMs and performed detailed classification of SCI patients using the cumulative distribution function (CDF). The optimal subtyping was achieved when the number of clusters (k) = 2, indicating the most appropriate number of patterns (**Figures 6A–C**). Therefore, SCI patients were divided into two subtypes: Subtype A and Subtype B. Furthermore, volcano plots and heatmaps illustrated the expression of differential mRNAs between the two subtypes (**Figures 6D, E**). Violin plots showed that the top 10 DEMs ranked by the absolute value of logFC, AHSP, ALAS2, CD177, HBG2, PCOLCE2, RHAG, RNY4, SELENBP1, SNORD88A, and WFDC1, exhibited significantly higher expression in Subtype A than in Subtype B within peripheral blood of SCI patients (**Figure 6F**).

### 3.7 Immune cell infiltration analysis

**Figure 7:**
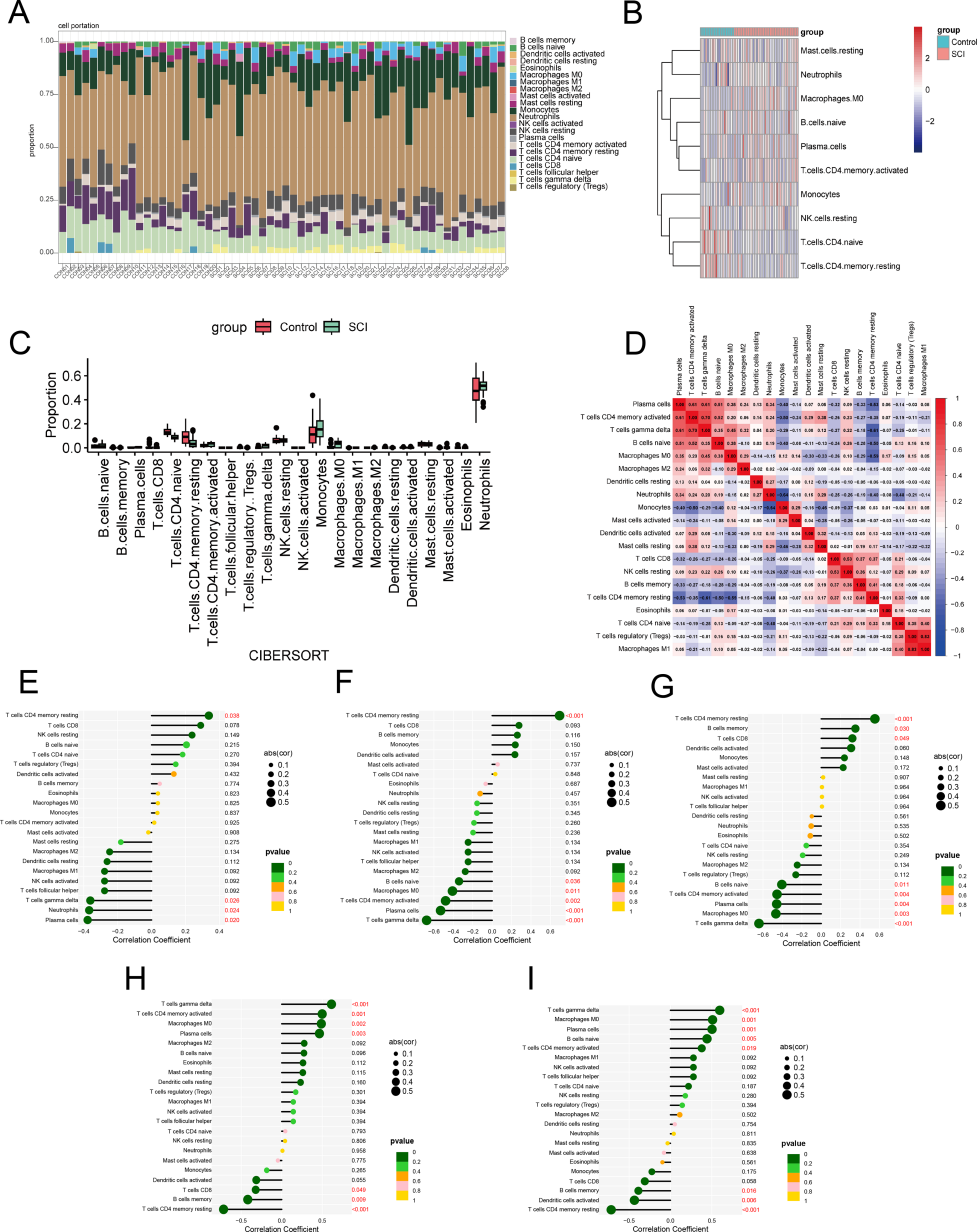
Immune cell infiltration. **(A)** Histogram of the percentage of 22 immunocyte subgroups in the control group and SCI group. **(B)** Heatmap of the expression levels of 22 immunocyte subgroups in SCI and control groups. **(C)** Box plot of the expression levels of 22 immunocyte subgroups in SCI and control groups. **(D)** Correlation degree of 22 immunocyte subgroups in every specimen. **(E)** The correlation between the infiltration of immune cells and BACH2 expression. **(F)** The correlation between the infiltration of immune cells and KLHL3 expression. **(G)** The correlation between the infiltration of immune cells and PRKCH expression. **(H)** The correlation between the infiltration of immune cells and MAP2K6 expression. **(I)** The correlation between the infiltration of immune cells and SIPA expression.

Subsequently, we employed the CIBERSORT algorithm to evaluate the degree of immune cell infiltration in SCI samples. The results showed that neutrophils and monocytes were highly abundant in both control samples and SCI samples (**Figure 7A**). This high baseline presence is attribute to the peripheral blood origin of the dataset. However, their abundances in SCI samples still higher than those in the control group. Compared with the control group, the proportions of CD4^+^ resting memory T cells and CD4^+^ naive T cells were significantly reduced in SCI samples (**Figure 7B**). In contrast, the proportions of macrophages M0, CD4^+^ activated memory T cells, γδ T cells and B cells naive were increased in SCI samples (**Figure 7C**). Additionally, the heatmap revealed that γδ T cells exhibited a positive correlation with CD4^+^ activated memory T cells, macrophages M0, and B cells naive, while showing a negative correlation with CD4^+^ resting memory T cells (**Figure 7D**). Compared with the control group, among the downregulated core mRNAs, we found that KLHL3, BACH2 and PRKCH exhibited a positive correlation only with CD4^+^ resting memory T cells, and the three genes showed a negative correlation with plasma cells and γδ T cells (**Figures 7E–G**). Additionally, KLHL3 and PRKCH were negatively correlated with B cells naive, Macrophages M0, and T cells CD4 memory activated (**Figures 7F, G**). Furthermore, BACH2 was also negatively correlated with neutrophils (**Figure 7E**), while PRKCH was positively correlated with B cells memory and T cells CD8 (**Figure 7G**). In the upregulated core mRNAs, we found that MAP2K6 and SIPA1L2 exhibited a positive correlation with T cells gamma delta, T cells CD4 memory activated, Macrophages M0, and Plasma cells, while the two genes showed a negative correlation with B cells memory and T cells CD4 memory resting (**Figures 7H, I**). Furthermore, MAP2K6 was also negatively correlated with T cells CD8 (**Figure 7H**), while SIPA1L2 was negatively correlated with Dendritic cells activated and positively correlated with B cells naive (**Figure 7I**).

### 3.8 Correlation between hub genes and immune cells

**Figure 8:**
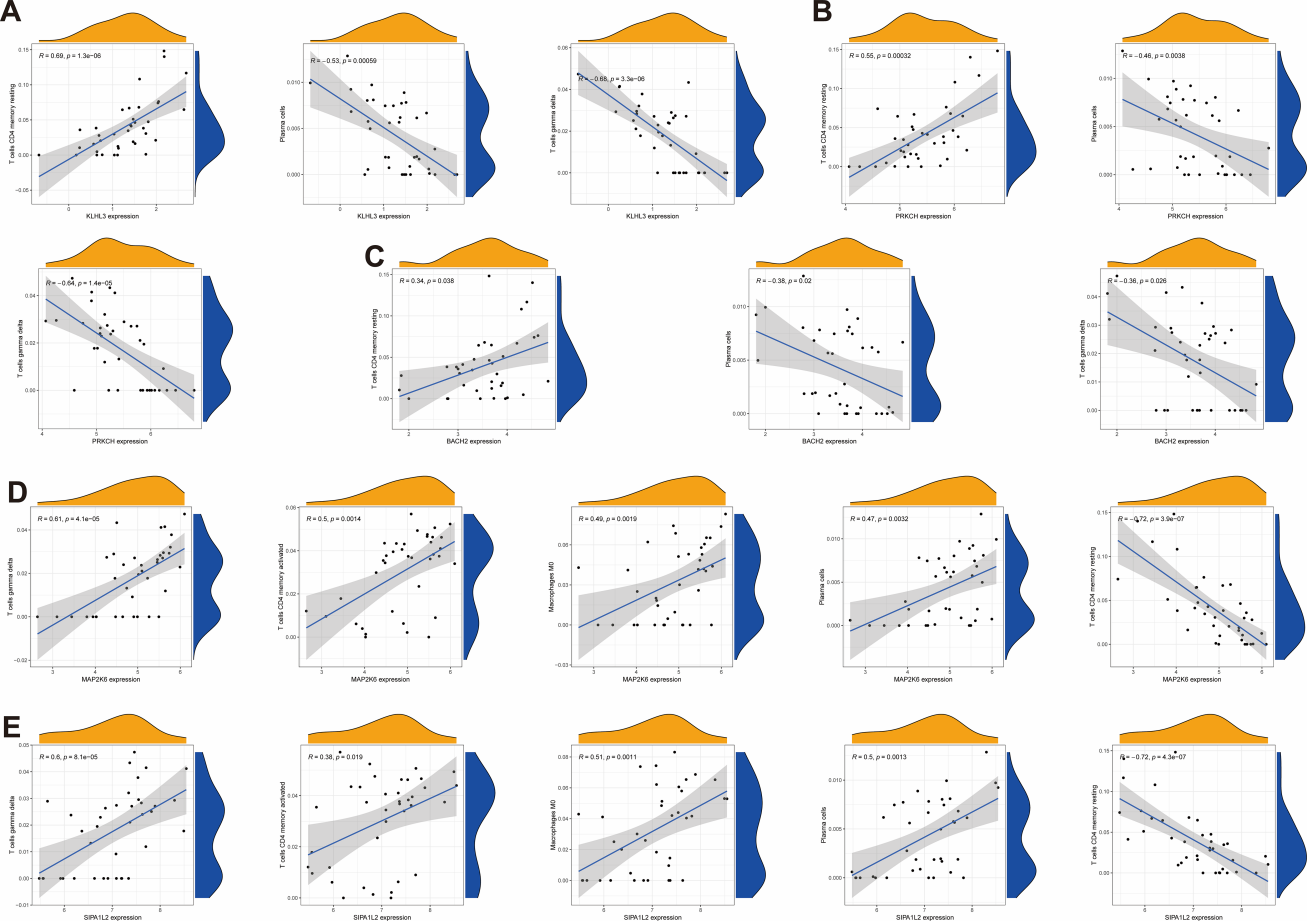
Spearman correlation analysis of hub mRNAs with immune cells. **(AI-AIII)** Spearman correlation analysis of KLHL3 with resting memory CD4^+^ T cells, Plasma cells and γδ T cells. **(BI-BIII)** Spearman correlation analysis of PRKCH with resting memory CD4^+^ T cells, Plasma cells and γδ T cells. **(CI-CIII)** Spearman correlation analysis of BACH2 with resting memory CD4^+^ T cells, Plasma cells and γδ T cells. **(DI-DV)** Spearman correlation analysis of MAP2K6 with γδ T cells, memory activated CD4^+^ T cells, M0 Macrophages, Plasma cells and memory resting CD4+ T cells. **(EI-EV)** Spearman correlation analysis of SIPA1L2 with γδ T cells, memory activated CD4^+^ T cells, M0 Macrophages, Plasma cells and memory resting CD4+ T cells.

Subsequently, we further analyzed the correlation between hub genes and immune cell infiltration levels to explore their potential roles in the progression of SCI. KLHL3, PRKCH, and BACH2 (**Figures 8A–C, I**), showed a positive correlation only with CD4^+^ resting memory T cells, with correlation coefficients of 0.69, 0.55 and 0.34, respectively. They (**Figures 8A–C, II-III**) also showed a negative correlation with plasma cells and γδ T cells, with correlation coefficients of -0.53, -0.46, and -0.38 for plasma cells, and -0.68, -0.64, and -0.36 for γδ T cells, respectively. Moreover, for BACH2, the correlation coefficients was -0.37 with neutrophils, for PRKCH, the correlation coefficients was 0.35 and 0.32 with B cells memory and T cells CD8 (**Supplementary 2A**). MAP2K6 and SIPA1L2 (**Figures 8D, E, I-IV**) showed a positive correlation with T cells gamma delta, T cells CD4 memory activated, Macrophages M0, and Plasma cells, with correlation coefficients of 0.61 and 0.6 for T cells gamma delta, 0.5 and 0.38 for T cells CD4 memory activated, 0.49 and 0.51 for Macrophages M0, and 0.47 and 0.5 for Plasma cells, respectively. They (**Figures 8D, E, V**) also showed a negative correlation with T cells CD4 memory resting and B cells memory, with correlation coefficients of -0.72 and -0.72 for T cells CD4 memory resting, and -0.42 and -0.39 for B cells memory, respectively. Moreover, the correlation coefficients of MAP2K6 were as follows: -0.32 with T cells CD8. For SIPA1L2, the correlation coefficients were -0.44 with Dendritic cells activated and 0.44 with B cells naive (**Supplementary 2B**). In conclusion, the downregulated KLHL3 and PRKCH showed strong positive correlations with resting T cells, whereas the upregulated MAP2K6 was positively correlated with activated immune cells and M0 macrophages, suggesting that these hub genes may participate in SCI-related immune inflammatory responses by modulating T cell activation and macrophage polarization.

### 3.9 Immune-associated co-expression modules identified by WGCNA

**Figure 9:**
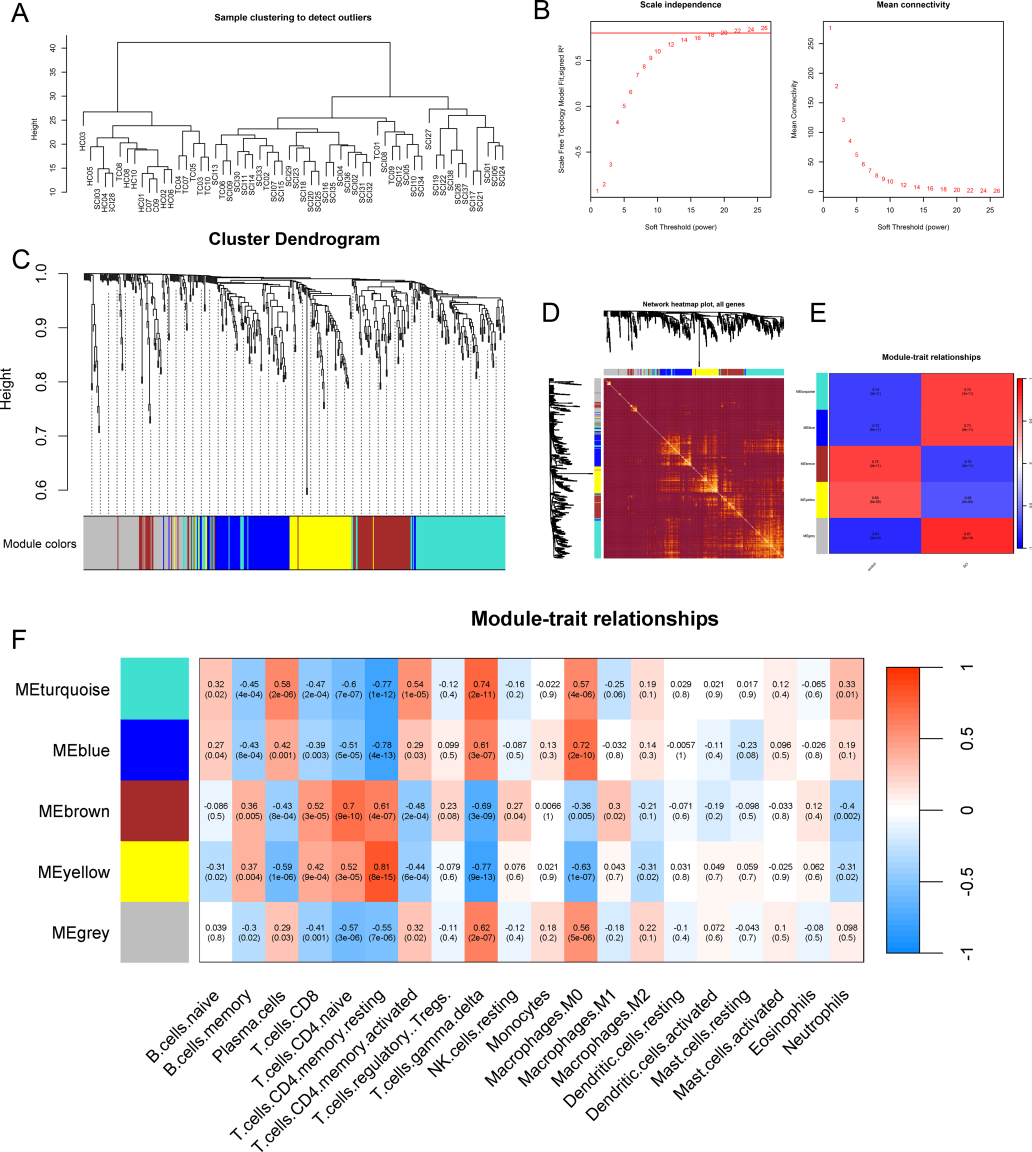
Construction of the co-expression network. **(A)** The sample dendrogram was drawn based on the Euclidean distance using the average clustering method for hierarchical clustering of samples, with each branch representing a sample, Height in the vertical coordinate being the clustering distance, and the horizontal coordinate being the clinical grouping information. **(B)** Soft threshold (power = 20) and scale-free topology fit index (R^2^ = 0.80). **(C)** Gene hierarchy tree-clustering Dendrogram. The graph indicates different genes horizontally and the uncorrelatedness between genes vertically, the lower the branch, the less uncorrelated the genes within the branch, the stronger the correlation. **(D)** The heatmap of correlations between modules. **(E)** The correlation of modules with SCI occurrence. **(F)** The correlation between the 20 types immune cells and modules, correlation coefficient and p-value are involved in modules.

To further delineate the immune cell types associated with the common target mRNAs, we constructed a gene co-expression network using the WGCNA algorithm. Hierarchical clustering of samples indicated good overall clustering without evident outliers (**Figure 9A**). A soft-thresholding power of 20 was selected to satisfy the scale-free topology criterion, yielding an R^2^ of 0.80 with relatively high mean connectivity (**Figure 9B**). Based on gene–gene correlations, a hierarchical clustering dendrogram was generated, from which we identified the yellow and turquoise modules, the former containing KLHL3, PRKCH and BACH2, and the latter containing SIPA1L2 (**Figure 9C**). The heatmap of correlations between modules is shown in **Figure 9D**. Five modules with significant correlation with SCI were identified (**Figure 9E**). Specifically, the MEturquoise, MEblue, and MEgrey modules were positively correlated with the SCI group, while the MEbrown and MEyellow modules were negatively correlated. The correlation between the 20 types immune cells and modules was visualized by heatmap (**Figure 9F**), We found that the yellow module exhibited a positive correlation with T cells CD4 memory resting (correlation coefficient of 0.81), while showing negative correlations with Plasma cells (-0.59), Macrophages M0 (-0.63), and T cells gamma delta (–0.77). In the turquoise module, there was a strong negative correlation with T cells CD4 memory resting (–0.77), whereas strong positive correlations were observed with Plasma cells (0.58), Macrophages M0 (0.57), T cells gamma delta (0.74), and T cells CD4 memory activated (0.54).

### 3.10 Gene enrichment analysis of functional modules

**Figure 10:**
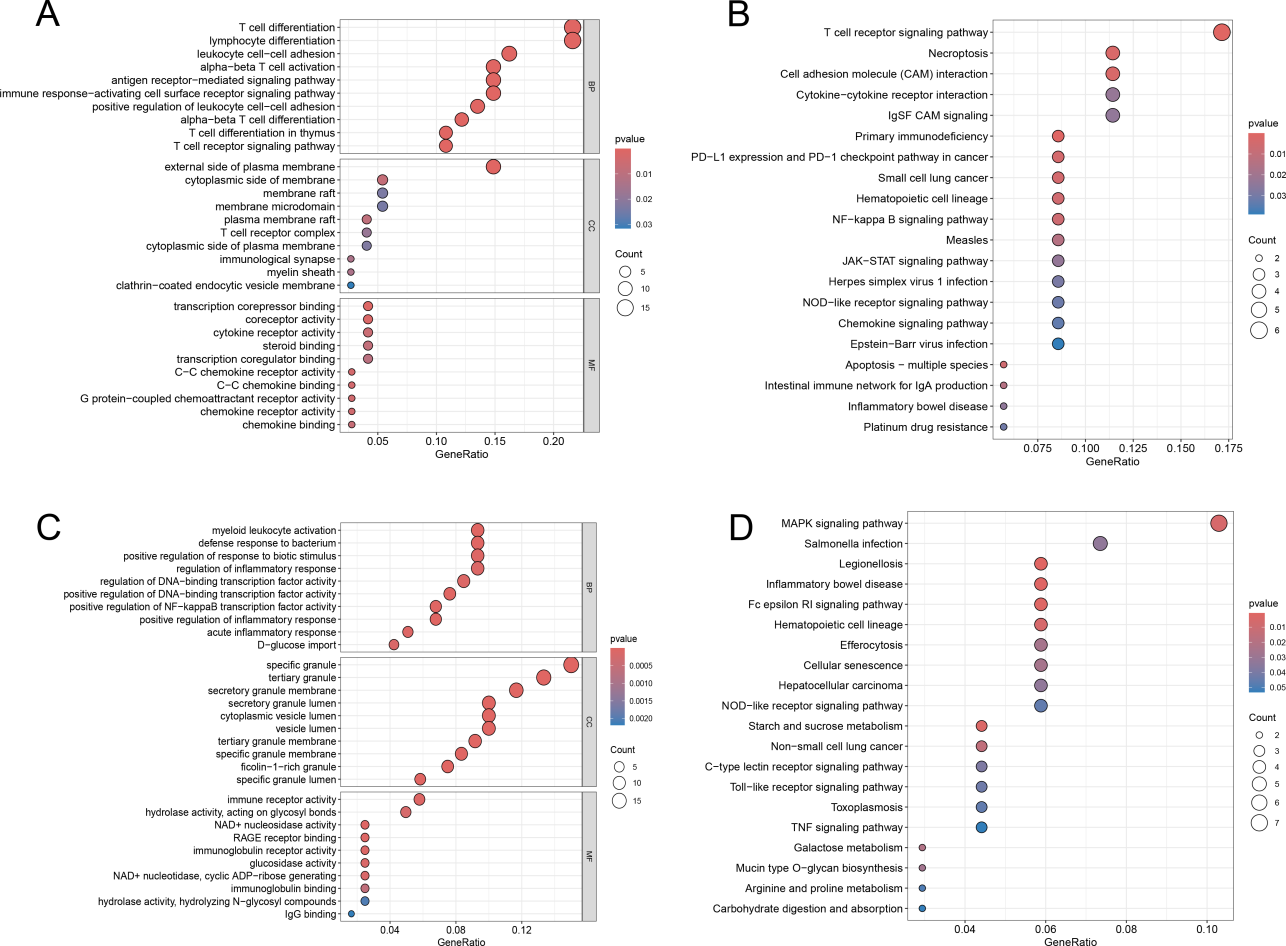
The GO and KEGG functional enrichment of mRNAs of modules. **(A)** The circle plot of GO enrichment of yellow module. **(B)** The bar plot of GO enrichment of yellow module. **(C)** The bar plot of KEGG enrichment of Turquoise module. **(D)** The bubble plot of KEGG enrichment of Turquoise module.

Based on the fact that the yellow and turquoise modules contain core mRNAs, GO and KEGG analyses of the two modules revealed distinct immune-related functions. The yellow module was mainly enriched in T cell differentiation and immune signaling pathways. biological process (BP) category of the GO terms (**Figure 10A**) included T cell differentiation, lymphocyte differentiation, and leukocyte cell-cell adhesion, while molecular function (MF) terms involved transcription corepressor binding, coreceptor activity, and cytokine receptor activity. KEGG analysis (**Figure 10B**) highlighted the T cell receptor signaling pathway, necroptosis, and cell adhesion molecule (CAM) interactions. Of note, hub genes like KLHL3, PRKCH, and BACH2 are not direct components of this pathway, and the enrichment primarily reflects the collective biological tendency of the co-expressed gene cluster. In contrast, the turquoise module was primarily associated with inflammatory and immune responses. BP terms (**Figure 10C**) were enriched for myeloid leukocyte activation, defense response to bacterium, and regulation of inflammatory response, with MF enrichment in immune receptor activity and hydrolase activity acting on glycosyl bonds. KEGG analysis (**Figure 10D**) identified the MAPK signaling pathway as a key enriched pathway. Specifically, analysis of the enriched genes, including MAP2K6, MAPK14, and GADD45A, indicated that the p38 MAPK branch is predominantly represented.

### 3.11 Molecular subtyping and immune correlations in SCI

**Figure 11:**
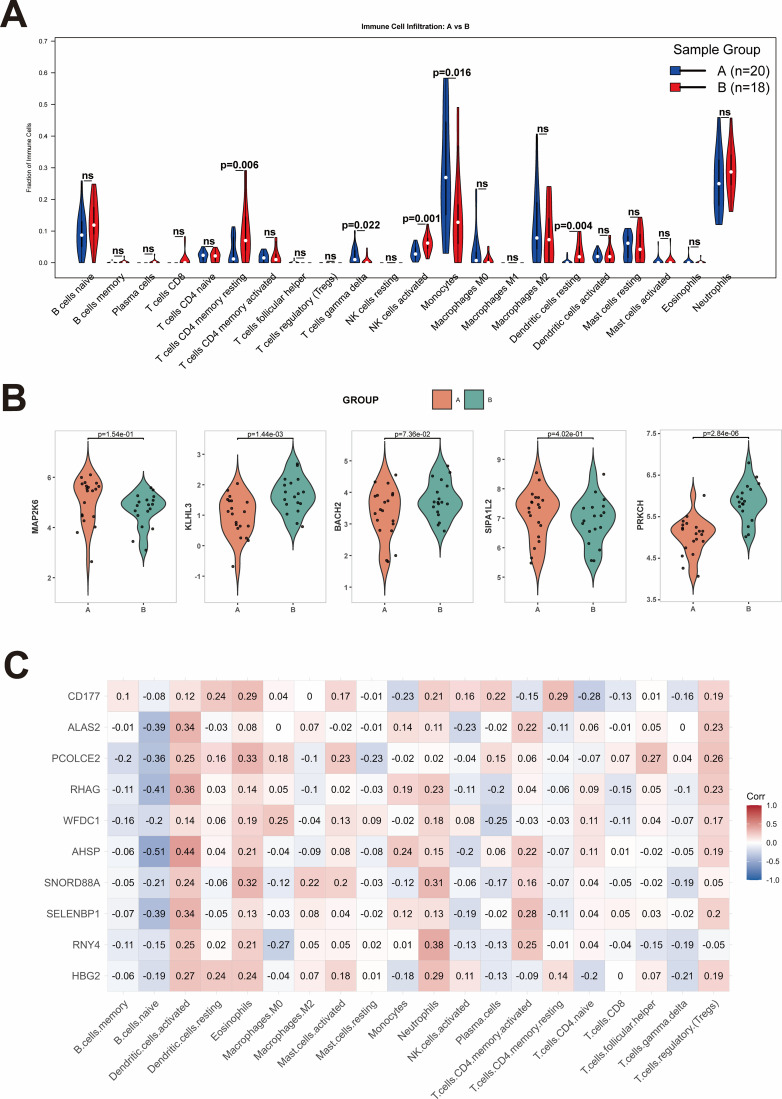
Correlation with immune cells of ten different mRNAs in SCI. **(A)** Violin plot of the expression levels of 22 immunocyte subgroups in A subtype and B subtype. **(B)** The expression of KLHL3, PRKCH, BACH2, MAP2K6 and SIPA1L2 in the A subtype and the B subtype. **(C)** Correlation Heatmap of ten different mRNAs across two subtypes.

We further explored the immune differences between Subtypes A and B in SCI. The results showed that the infiltration of monocytes was significantly higher in Subtype A than in Subtype B (p = 0.016). In contrast, the infiltration of CD4^+^ resting memory T cells (p = 0.006), NK cells activated (p = 0.001), and dendritic cells resting (p = 0.004) was significantly higher in Subtype B than in Subtype A. Although the infiltration of B cells naive and neutrophils was also higher in Subtype B than in Subtype A, these differences were not statistically significant (**Figure 11A**). Compared to Subtype A, Subtype B showed slightly higher expression of KLHL3, PRKCH, and BACH2, but lower expression of MAP2K6 and SIPA1L2 (**Figure 11B**). After screening the top 10 differential mRNAs between the two subtypes, we performed correlation analysis between these mRNAs and immune cells. The results revealed that nearly all of these differential mRNAs exhibited a positive correlation with neutrophils, dendritic cells activated, and eosinophils, while showing a negative correlation with B cells naive (**Figure 11C**).

### 3.12 Validation of core miRNA *in vivo*

**Figure 12:**
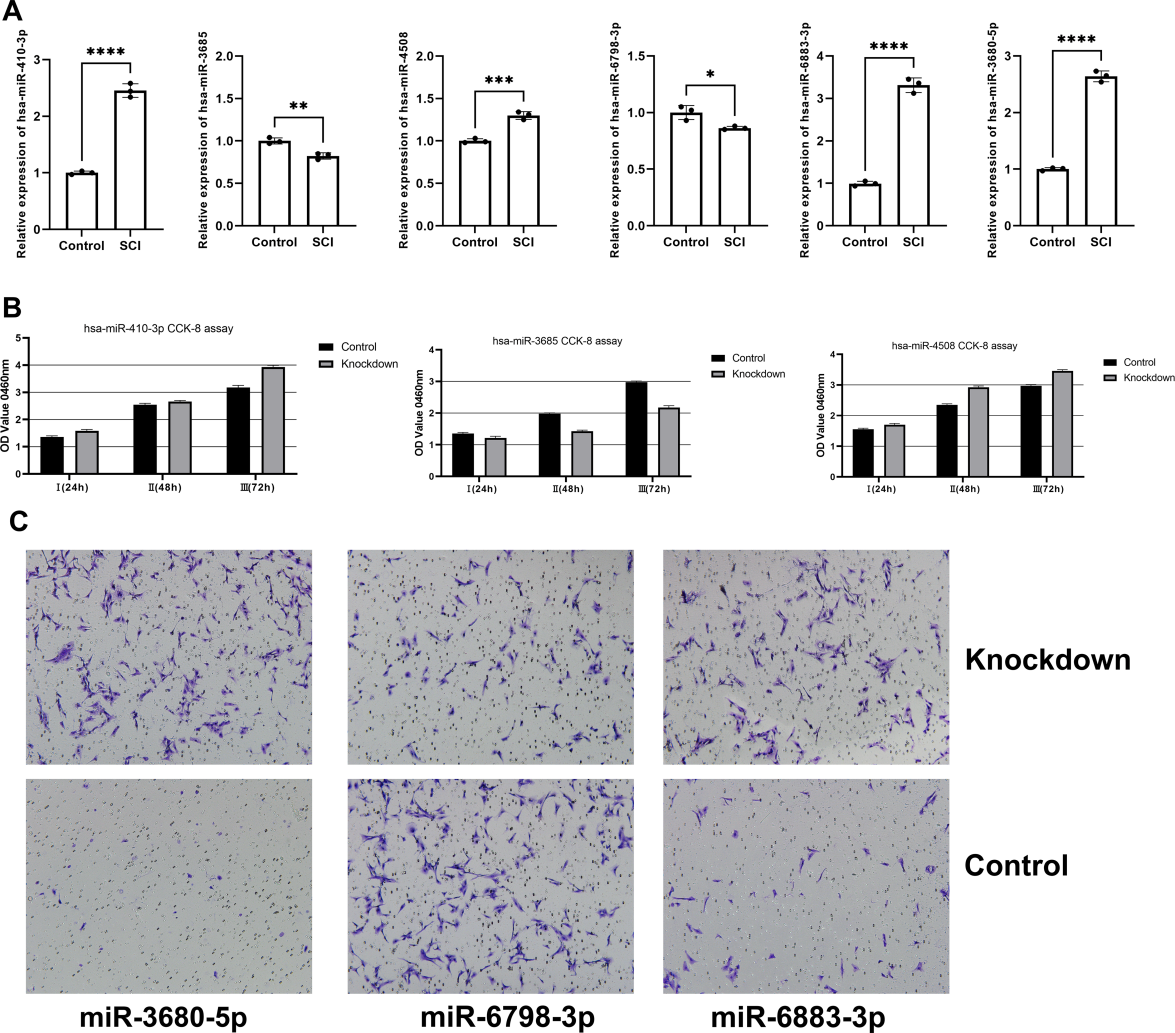
The validation of six core miRNAs *in vitro*. **(A)** RT-qPCR levels in SCI patients and corresponding control groups. **(B)** The CCK-8 assay. **(C)** Transwell assay. * (p < 0.05), ** (p < 0.01) , *** (p < 0.001), **** (p < 0.0001).

Finally, RT-qPCR was employed to validate the expression of six core miRNAs in the blood of patients with SCI. Compared to healthy controls, significant dysregulation was observed in all six miRNAs. Specifically, hsa-miR-3680-5p, hsa-miR-6883-3p, hsa-miR-410-3p, and hsa-miR-4508 were significantly upregulated. In contrast, hsa-miR-3685 and hsa-miR-6798-3p were found to be significantly downregulated (**Figure 12A**). Furthermore, we respectively knockdowned the six core miRNAs in C8-D1A astrocyte cell line obtained from Shanghai Jinyuan Biotechnology Co., Ltd. The subsequent CCK-8 assay indicated that the expression levels of miR-410-3p, miR-3685, and miR-4508 were closely associated with C8-D1A cell proliferation (**Figure 12B**), while knockdown of miR-3680-5p, miR-6798-3p, and miR-6883-3p can’t affect the proliferation activity of C8-D1A cells. To further investigate whether miR-3680-5p, miR-6798-3p, and miR-6883-3p influence astrocytes involved in tissue repair through other mechanisms in SCI, we performed transwell assays. The results showed that when co-cultured with macrophages, knockdown of these genes significantly affected the cell migration ability of C8-D1A cells (**Figure 12C**).

## 4 Discussion

As an important gene expression regulatory pattern discovered in recent years, the ceRNA network provides a novel research perspective for deciphering the pathophysiological processes of SCI, identifying potential therapeutic targets, and developing new therapeutic strategies. In recent years, a growing number of studies have screened the core nodes of the ceRNA network in SCI (14). However, the specific regulatory mechanisms within the ceRNA network remain insufficiently clarified, and most studies have been limited to mouse models. Therefore, based on the sequencing results, we employed bioinformatics analysis to identify potential SCI-associated regulatory nodes, construct a core ceRNA network, and further investigate the impact of immune cell infiltration on SCI.

This study conducted miRNA sequencing on blood samples from 6 spinal cord injury patients and six healthy volunteers, along with screening of the GSE151371 dataset to identify DEmiRNAs and DEMs between SCI and normal samples. Preliminary findings revealed associations between these DEmiRNAs and pathways related to inflammation and the immune system. We further confirmed six core miRNAs, hsa-miR-3680-5p, hsa-miR-410-3p, hsa-miR-6883-3p, hsa-miR-3685, hsa-miR-4508 and hsa-miR-6798-3p, and their shared targets KLHL3, BACH2, PRKCH, MAP2K6 and SIPA1L2. Additionally, we predicted lncRNAs associated with hsa-miR-3685 and hsa-miR-410-3p, constructing a core ceRNA network for SCI. The study classified SCI into two molecular subtypes (Type A and Type B), identifying their respective DEMs. Immune infiltration and cellular correlation analyses revealed significant infiltration of neutrophils and monocytes in SCI, while T cells, B cells, and macrophages showed reduced infiltration. Notably, KLHL3, BACH2 and PRKCH exhibited positive correlations with T cells CD4 memory resting, but negative correlations with γδ T cells and plasma cells. Besides, MAP2K6 and SIPA1L2 also exhibited positive correlations with γδ T cells, T cells CD4 memory activated, Macrophages M0 and Plasma cells, but negative correlations with T cells CD4 memory resting. The research also explored immune differences between Type A and Type B subtypes in SCI. Furthermore, through validation of patient and healthy population data, we confirmed abnormally high expression of core miRNAs hsa-miR-4508, hsa-miR-3680-5p, hsa-miR-410-3p, and hsa-miR-6883-3p. These core miRNAs can be distinguished in the peripheral blood of patients with SCI compared to healthy controls. These findings integrate ceRNA regulatory networks with immune cell infiltration and related functional pathways, providing new insights into understanding spinal cord injury pathogenesis and developing targeted drug or gene therapy strategies for ceRNA networks. To experimentally validate these findings, we performed RT-qPCR to examine the expression levels of the six core miRNAs in peripheral blood from SCI patients and healthy controls. The results confirmed that hsa-miR-4508, hsa-miR-3680-5p, hsa-miR-410-3p, hsa-miR-3685, miR-6798-3p, and hsa-miR-6883-3p were significantly aberrantly expressed in SCI patients, consistent with sequencing data. These core miRNAs effectively distinguished SCI patients from healthy controls, suggesting their potential as diagnostic biomarkers. Furthermore, to investigate the functional roles of the core miRNAs in astrocyte-mediated tissue repair following spinal cord injury (SCI), we performed knockdown of six core miRNAs and assessed cell proliferation viability using CCK-8 assays. The results showed that three miRNAs (hsa-miR-410-3p, hsa-miR-4508, and hsa-miR-3685) directly affected astrocyte proliferation, whereas the other three (miR-3680-5p, miR-6798-3p, and miR-6883-3p) did not. To further explore how these three miRNAs, which did not directly affect viability, contribute to SCI pathology, we conducted Transwell co-culture assays with macrophages for these three miRNAs. The results revealed that upon co-culture with macrophages, knockdown of these three miRNAs significantly altered the migratory capacity of C8-D1A astrocytes. Collectively, these findings suggest that miR-3680-5p, miR-6798-3p, and miR-6883-3p may not directly regulate astrocyte proliferation but instead modulate astrocyte-macrophage crosstalk, thereby influencing astrocyte migration within the injured spinal cord microenvironment and contributing to tissue repair. These results indicate that miR-3680-5p, miR-6798-3p, and miR-6883-3p may not directly modulate astrocyte proliferation but rather influence tissue repair by regulating astrocyte-macrophage crosstalk, thereby affecting astrocyte migration in the injured spinal cord microenvironment.

MiRNAs are a class of non-coding RNAs that represent a central post-transcriptional regulatory system by inhibiting translation initiation or targeting transcripts for sequestration or degradation. MiRNAs have been identified as highly stable in biological fluids (15). These single-stranded RNAs, 19–25 nucleotides in length, are abundant in the central nervous system and contribute to various neuronal processes, such as synaptic development, maturation, and plasticity (16, 17). Consistent with our initial sequencing data, RT-qPCR validation confirmed the significant dysregulation of multiple candidate miRNAs in SCI patients. Specifically, hsa-miR-3680-5p and hsa-miR-6883-3p were significantly upregulated, whereas hsa-miR-6798-3p was downregulated in the SCI group compared to healthy controls. Given their prominent roles in regulating astrocyte-macrophage crosstalk as demonstrated in our functional assays, we specifically focused on miR-3680-5p, miR-6798-3p, and miR-6883-3p. Previous studies on hsa-miR-3680-5p have shown its high expression in patients with bipolar disorder (BD), where it may be associated with low levels of the MAOA enzyme (18), suggesting its potential involvement in central nervous system pathologies. Furthermore, a study by Chen et al. (19) demonstrated that miR-6798-3p regulates cell proliferation and apoptosis via the p53/p21 pathway in glioblastoma, indicating its active participation in the central nervous system microenvironment. Similarly, Hao et al. (20) demonstrated that miR-6883-3p acts as a critical regulator of cell growth and immune responses by directly targeting the Toll/interleukin-1 receptor domain-containing adaptor protein (TIRAP). Given that TIRAP-mediated Toll-like receptor signaling is fundamental to macrophage activation and neuroinflammation, this mechanism provides a compelling molecular basis for our observation that miR-6883-3p specifically influences astrocyte-macrophage crosstalk in the SCI microenvironment. Therefore, above miRNAs are expected to become new targets for the treatment and rehabilitation of SCI.

In our study, KLHL3, PRKCH, BACH2, MAP2K6 and SIPA1L2 are the common targets of six core miRNAs. In SCI, KLHL3, PRKCH and BACH2 are downregulated when the expression of miRNA increases, and participate in regulating the progression of SCI. KLHL3 belongs to the Kelch-like (KLHL) gene family. The 42-member KLHL protein family serves as adaptors for ubiquitin E3 ligase complexes, controlling the stability of various substrates. KLHL proteins are essential for maintaining protein homeostasis in multiple tissues and are mutated in human diseases, including neurodegenerative diseases as the predominant neurological disorders (21). In the event of a spinal cord injury, excessive excitation of spinal reflexes and reduced synaptic inhibition are typically associated with spasticity following SCI. In adults, due to the low intracellular chloride (Cl^-^) concentration, activation of γ-aminobutyric acid type A (GABAA) and glycine receptors inhibits neurons, and the intracellular chloride (Cl^-^) concentration is maintained by the potassium-chloride cotransporter KCC2 (encoded by Slc12a5). KLHL3 and its downstream molecule WNK may regulate neuronal excitability by modulating KCC2. In our study, we found that the expression level of KLHL3 is significantly reduced in the peripheral blood of SCI patients. Although the aforementioned mechanistic studies primarily focus on local processes within central nervous system neurons, peripheral blood serves as a critical systemic window reflecting the profound neuro-immune interactions following SCI (22). The systemic downregulation of KLHL3 in peripheral circulation may mirror the severe pathological stress and inflammatory cascade originating from the injured spinal cord. Furthermore, considering that circulating immune cells infiltrate the lesion site through the compromised blood-spinal cord barrier (23, 24), altered KLHL3 expression in peripheral blood may also directly participate in regulating the systemic immune response and shaping the neuroinflammatory microenvironment after injury. BACH2 is a member of the Bach family and belongs to the BTB-basic leucine zipper (bZip) transcription factor family (25). As a super-enhancer and transcriptional repressor, BACH2 regulates the differentiation and maturation of Th2-associated immune cells, including those of the B cell lineage and T cell lineage (26). In humans, genome-wide association studies have linked polymorphisms in the BACH2 locus to autoimmune and inflammatory diseases (27), and preclinical models have associated BACH2 with the regulation of CD4^+^ T cells, indicating that it can prevent the induction of excessive inflammation (28). Tsukumo S et al. (29) proposed that the calcium-binding protein S100A is a putative direct target of BACH2. They found that the expression of S100a1 was increased in Th17iv cells compared with naive T cells, and this expression was negatively correlated with Bach2 expression. S100A has been suggested to promote inflammation and autoimmunity, as its expression is associated with the severity of inflammatory diseases such as rheumatoid arthritis and atherosclerosis (30). Therefore, the observed downregulation of BACH2 combined with elevated S100a1 levels is likely a relevant feature of autoimmune neuroinflammation. Moreover, studies have shown that miR-mediated downregulation of BACH2 is associated with increased susceptibility to cell death in different cell types (31). In our study, BACH2 expression was significantly downregulated in peripheral blood of SCI patients, which is mediated by hsa-miR-4508, hsa-miR-6883-3p, hsa-miR-3680-5p and hsa-miR-410-3p. We also found that the BACH2 high-expression group was enriched in the T cell receptor signaling pathway and B cell receptor signaling pathway. Consistent with these findings, in Experimental Autoimmune Encephalomyelitis (EAE), BACH2 is proposed to prevent inflammatory disease such as Multiple Sclerosis (MS) by maintaining immune tolerance and regulating CD4^+^ T−cell differentiation (32). Thus, the low expression of BACH2 in peripheral blood of SCI patients may be involved in inhibiting the differentiation of T cells and B cells, as well as enhancing susceptibility to autoimmunity and cell death, thereby contributing to the progression of SCI. PRKCH (PKC-η) belongs to the protein kinase C (PKC) family and is involved in regulating cell proliferation, differentiation, and death. Wang et al. found that knockdown of PKC-η significantly reduced IL-1β and TNF-α levels in BV2 cells and astrocytes, indicating its involvement in neuroinflammation. Furthermore, detection of NF-κB pathway-related proteins following PKC-β knockdown revealed that si-PKC-η downregulated phosphorylated p65 and p-IκBα levels, demonstrating that PKC-η mediates NF-κB-regulated inflammatory responses (33). In our study, PRKCH expression was downregulated in peripheral blood of SCI patients, which is mediated by hsa-miR-4508, hsa-miR-6883-3p, hsa-miR-3680-5p and hsa-miR-410-3p. MAP2K6, a member of the MAPK kinase family, primarily activates the p38/MAPK pathway and is involved in regulating physiological processes such as inflammation, stress response, cell death, and metabolic balance (34). Its activation promotes NF-κB nuclear translocation and the release of pro-inflammatory factors, such as TNF-α, and IL-6, in a kinase activity-dependent manner, thereby amplifying inflammatory responses. In the study by Sheng et al. (35), Quercetin (QE) significantly upregulates miR-216b, which directly targets and binds to MAP2K6 mRNA, thereby suppressing its expression. As a key upstream kinase of the p38 pathway, the downregulation of MAP2K6 directly attenuates the activation of the entire p38/MAPK signaling cascade. In our study, PRKCH expression was upregulated in peripheral blood of SCI patients, which is mediated by hsa-miR-3685 and hsa-miR-6798-3p. This indicates that MAP2K6 may be a potential therapeutic target for SCI. SIPA1L2 known as SPAR2, a member of the SIPA1L family of neuronal RapGAPs, regulates Rap1. This protein is most abundant in granule cells of the dentate gyrus and cerebellum and exhibits RapGAP activity toward Rap1/2. By enhancing the intrinsic GTPase activity of Rap1/2, it catalyzes the hydrolysis of GTP to GDP, thereby inactivating Rap1/2 and subsequently attenuating ERK signaling. SIPA1L2 interacts with Light Chain 3 (LC3), an autophagosome marker involved in substrate selection. This interaction promotes the RapGAP activity of SIPA1L2 and enhances autophagy (36). In our study, SIPA1L2 expression was upregulated in peripheral blood of SCI patients, which is mediated by hsa-miR-3685 and hsa-miR-6798-3p.

In the correlation analysis between KLHL3, PRKCH, BACH2 and immune cells, we observed a significant negative correlation with γδ T cells and these three hub mRNAs. γδ T cells are T lymphocytes that express the γ and δ chains of the T cell receptor (TCR) to form the γδ T cell receptor (γδ TCR). γδ T cells are a relatively small subset of T lymphocytes in peripheral blood (PB), accounting for only 1–5% of circulating lymphocytes (37). Despite their small numbers, their role in immunity is not to be underestimated. Their activation process is faster than that of conventional T cells, and they secrete interleukin-17 (IL-17), which is a crucial regulatory molecule that acts as a potent early inflammatory factor in related diseases. Additionally, γδ T cells can activate B cells, dendritic cells (DCs), αβ T cells, and natural killer (NK) cells through multiple mechanisms, thereby promoting inflammation. Sustained neuroinflammation leads to chronic inflammation and neuronal death. As initiators of inflammation, γδ T cells are involved in many neuroinflammation-related diseases (38). Studies have shown that the immune response induced by γδ T cells is closely associated with SCI-related neuroinflammation (39). The recruitment of γδ T cells to the SCI site promotes inflammatory responses and exacerbates nerve damage.γδ T cells, particularly Vγ4+γδ T cells, may contribute to the aggravation of SCI lesions by serving as a key source of interferon-γ (IFN-γ), inducing macrophages to adopt the M1 phenotype, and increasing the secretion of inflammatory cytokines such as tumor necrosis factor-α (TNF-α) (40, 41). Xu et al. (42) proposed that γδ T cells and IL-17 are associated with SCI: γδ T cells can be recruited to the SCI site via C-C motif chemokine ligand 2/C-C chemokine receptor type 2 (CCL2/CCR2) signaling, thereby promoting inflammatory responses and worsening nerve injury. In a study by Liu et al. (43), it was found that the frequency of IL-17+γδ T cells in the spinal cords of rats changed after short-chain fatty acid (SCFA) treatment; thus, IL-17+γδ T cells were identified as potential effector T cells. Our study also confirms these findings: we observed that the infiltration of γδ T cells was higher in the SCI group than in the control group. Furthermore, KLHL3, PRKCH and BACH2, the three hub mRNAs we identified, were shown to have a significant negative correlation with γδ T cells. Instead, given that our results are based on correlation analyses, these findings should be interpreted as indicating an association rather than a definitive causal relationship. We propose a tentative hypothesis that reduced KLHL3, PRKCH and BACH2 expression may be associated with increased γδ T cell infiltration in SCI, potentially contributing to the inflammatory microenvironment. In addition, we also found that plasma cells and M0 macrophages were positively correlated with MAR2K6 and SIPA1L2. In the study by Daniel P Ankeny et al., Plasma cells were found in the spinal cord of mice with SCI, where they seem to produce deleterious antibodies that compromise functional recovery (44). M0 macrophages are inactivated macrophages that can polarize into pro-inflammatory M1 macrophages when stimulated by TNF-α, LPS, and IFN-γ, or differentiate into M2 macrophages under the influence of IL-4, IL-13, IL-10, and IL-21 (45). In SCI, increased M0 macrophages may exert a bidirectional regulatory effect. Crucially, the polarization state and spatial dynamics of these macrophages directly dictate the behavior of adjacent astrocytes. A study by Haan et al. (46) demonstrated that the crosstalk between infiltrating macrophages and astrocytes significantly alters astrocyte reactivity and the broader inflammatory response during reactive gliosis. Furthermore, regarding the spatial reorganization of the glial scar, Ono et al. (47) revealed that macrophages play a leading role in determining the direction of astrocytic migration toward the lesion center via the ADP-P2Y1R axis. These mechanisms strongly contextualize our functional findings: by modulating astrocyte-macrophage crosstalk, the miR-3680-5p, miR-6798-3p, and miR-6883-3p network likely influences the microenvironmental macrophage status, which in turn critically guides astrocyte migration and subsequent tissue repair in the injured spinal cord.

In our study on Subtypes A and B of SCI, we found that the infiltration of neutrophils, monocytes, and activated dendritic cells was more prominent in Subtype A. Studies have shown that natural killer (NK) cells are activated within 24 hours after traumatic SCI (48). Inhibiting the recruitment of peripheral neutrophils and monocytes can reduce oxidative stress associated with non-heme iron accumulation in lesions. Under such conditions, the long-term neurological function recovery (28–42 days) after SCI surgery is significantly improved (49). This suggests that Subtype A identified in our study may be in the acute phase of SCI with more severe injury.

Meanwhile, we observed that the infiltration of resting CD4^+^ memory T cells, activated NK cells, and resting dendritic cells was more significant in Subtype B. Notably, the most important immunosuppression after SCI is a marked reduction in the frequency and function of T cells and NK cells, which is affected by the severity of injury (50). Studies have indicated that in patients with (sub)acute SCI, the distribution of CD4^+^ T cells shifts from more naive T cells to more memory T cells (51). We also observed that the expression level of KLHL3, PRKCH and BACH2 in Subtype B were higher than that in Subtype A, and we previously confirmed that KLHL3, PRKCH and BACH2 has a positive correlation with resting CD4^+^ memory T cells. These findings suggest that Subtype B identified in our study may be in the subacute or chronic phase of injury.

LncRNAs and circRNAs can competitively bind to miRNAs and their miRNA response elements (MREs). Acting as ceRNAs, they regulate the expression levels of miRNAs that target mRNAs (52). In microarray or sequencing studies, specific miRNAs (miR-21, miR-486, and miR-20) are dysregulated and associated with SCI by modulating inflammation, gliosis, cell death, or regeneration (53). Previous studies also support that the lncRNA/circRNA-miRNA-mRNA interaction can be regarded as a crucial mechanism for the development and initiation of SCI (54, 55). In this study, we analyzed and identified the core-expressed miRNAs. By predicting their corresponding lncRNAs and target mRNAs, we constructed a ceRNA network based on lncRNA/circRNA-miRNA-mRNA interactions. Furthermore, we found that two lncRNAs, MIR17HG and XIST, are common lncRNAs targeting the core miRNAs hsa-miR-3685 and hsa-miR-410-3p. Zhang et al.’s study find that MIR17HG is highly expressed in Parkinson’s disease (PD) tissues and cells. Reducing its expression can decrease neuronal apoptosis, microglial inflammation, and oxidative stress in PD models, and it regulates PD progression by modulating the miR-153-3p/SNCA axis (56). Another study observed that XIST and Smurf1 levels were higher in primary microglia induced by SCI rats and LPS. Knocking down XIST could reduce apoptosis and inflammatory damage in LPS-induced microglia and SCI rats by directly regulating the miR-27a/Smurf1 axis (57). This strongly validates our findings and suggests that these lncRNAs or circRNAs may play a central role in the entire ceRNA network, participating in the regulation of core miRNA and hub mRNA, thereby promoting SCI occurrence and progression.

However, our study has limitations. Specifically, the miRNA and mRNA expression data were derived from different sample sources, which prevented us from performing integrated correlation or mediation analyses to explore the specific regulatory mechanisms between lncRNAs/circRNA, miRNAs, and mRNAs in detail. Future research should utilize paired multi-omics datasets to explore and refine the ceRNA network in SCI more deeply and further validate potential targets that can be translated into clinical therapies.

## 5 Conclusion

In conclusion, this study constructs a comprehensive, human-specific ceRNA regulatory network associated with the immune microenvironment of SCI. We identified a distinct set of core miRNAs (including hsa-miR-4508 and hsa-miR-3685) and established their potential regulatory links with key immune-related mRNAs such as KLHL3 and MAP2K6. Crucially, our bioinformatic analysis suggests that these ceRNA axes do not function in isolation but likely orchestrate a shift in the immune landscape, suppressing protective T cell responses while promoting pro-inflammatory infiltration. Unlike prevalent animal-based models, our findings are derived from clinical blood samples, offering a more translationally relevant perspective on the human SCI pathology. Looking forward, future studies should focus on two key directions: first, experimentally validating the direct physical interactions of these predicted axes using luciferase reporter assays; and second, evaluating the therapeutic potential of targeting these core miRNAs to modulate immune responses in diverse *in vivo* models.

## Data Availability

The raw data supporting the conclusions of this article will be made available by the corresponding authors on request.
